# 4-(1-Methyl­eth­yl)-*N*-((*E*)-4-{[1-(prop-2-en-1-yl)-1*H*-1,2,3-triazol-4-yl]meth­oxy}benzyl­idene)aniline

**DOI:** 10.1107/S1600536813000755

**Published:** 2013-01-19

**Authors:** Mehmet Akkurt, Aliasghar Jarrahpour, Mehdi Mohammadi Chermahini, Pezhman Shiri, Muhammad Nawaz Tahir

**Affiliations:** a Department of Physics, Faculty of Sciences, Erciyes University, 38039 Kayseri, Turkey; b Department of Chemistry, College of Sciences, Shiraz University, 71454 Shiraz, Iran; c Department of Physics, University of Sargodha, Sargodha, Pakistan

## Abstract

In the title compound, C_22_H_24_N_4_O, the terminal and central benzene rings make dihedral angles of 52.7 (3) and 43.8 (2)°, respectively, with the triazole ring. The dihedral angle between the benzene rings is 8.9 (2)°. The crystal structure features C—H⋯π inter­actions. The atoms of the terminal propenyl group are disordered over two sets of sites, with a refined occupancy ratio of 0.714 (14):0.286 (14).

## Related literature

For bond-length data, see: Allen *et al.* (1987 ▶). For general background to the properties of Schiff bases, see: Ajello & Cusmanos (1940 ▶); Dhar & Taploo (1982 ▶); Holla *et al.* (2005 ▶); Singh *et al.* (2012 ▶); Supuran *et al.* (1996 ▶).
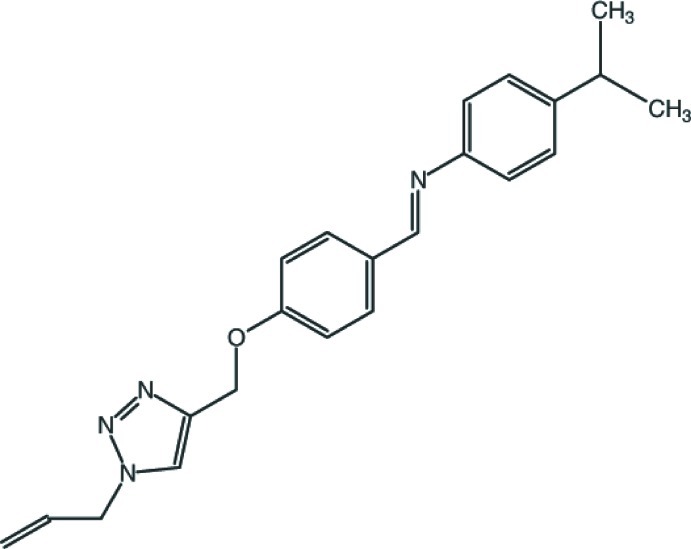


## Experimental

### 

#### Crystal data

C_22_H_24_N_4_O
*M* *_r_* = 360.45
Monoclinic, 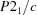

*a* = 5.5885 (11) Å
*b* = 8.3929 (18) Å
*c* = 42.069 (9) Å
β = 92.149 (10)°
*V* = 1971.8 (7) Å^3^
*Z* = 4
Mo *K*α radiation
μ = 0.08 mm^−1^
*T* = 296 K
0.30 × 0.20 × 0.18 mm

#### Data collection

Bruker Kappa APEXII CCD diffractometer
Absorption correction: multi-scan (*SADABS*; Bruker, 2005 ▶) *T*_min_ = 0.982, *T*_max_ = 0.986
13866 measured reflections
3451 independent reflections
1259 reflections with *I* > 2σ(*I*)
*R*_int_ = 0.082

#### Refinement

*R*[*F* ^2^ > 2σ(*F* ^2^)] = 0.068
*wR*(*F* ^2^) = 0.217
*S* = 0.96
3451 reflections
256 parameters
7 restraints
H-atom parameters constrained
Δρ_max_ = 0.20 e Å^−3^
Δρ_min_ = −0.20 e Å^−3^

### 

Data collection: *APEX2* (Bruker, 2009 ▶); cell refinement: *SAINT* (Bruker, 2009 ▶); data reduction: *SAINT*; program(s) used to solve structure: *SHELXS97* (Sheldrick, 2008 ▶); program(s) used to refine structure: *SHELXL97* (Sheldrick, 2008 ▶); molecular graphics: *ORTEP-3 for Windows* (Farrugia, 2012 ▶) and *PLATON* (Spek, 2009 ▶); software used to prepare material for publication: *WinGX* (Farrugia, 2012 ▶) and *PLATON*.

## Supplementary Material

Crystal structure: contains datablock(s) global, I. DOI: 10.1107/S1600536813000755/bq2382sup1.cif

Structure factors: contains datablock(s) I. DOI: 10.1107/S1600536813000755/bq2382Isup2.hkl

Supplementary material file. DOI: 10.1107/S1600536813000755/bq2382Isup3.cml

Additional supplementary materials:  crystallographic information; 3D view; checkCIF report

## Figures and Tables

**Table 1:** Hydrogen-bond geometry (Å, °) *Cg*1 and *Cg*3 are the centroids of the N2–N4/C18/C19 1H-1,2,3-triazole and C11–C16 benzene rings, respectively.

*D*—H⋯*A*	*D*—H	H⋯*A*	*D*⋯*A*	*D*—H⋯*A*
C2—H2⋯*Cg*3^i^	0.93	2.96	3.752 (5)	144
C8—H8*A*⋯*Cg*1^ii^	0.96	2.80	3.678 (6)	153

Symmetry codes: (i) 
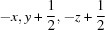
; (ii) 
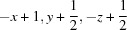
.

**Fig. 1.**
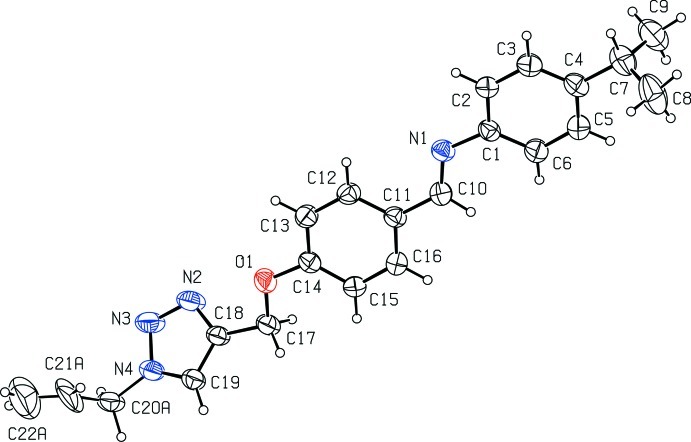
ORTEP drawing of the title compound with atomic numbering scheme and thermal ellipsoids at 30% probability level. The minor disorder component is not shown.

## supplementary crystallographic information

### Comment

Compounds containing an azomethine group (–CH=N–), known as Schiff bases are formed by the condensation of a primary amine with a carbonyl compound. Schiff bases are some of the most widely used organic compounds. They are used as pigments and dyes, catalysts, intermediates in organic synthesis, and as polymer stabilisers (Dhar & Taploo, 1982). In azomethine derivatives, the C=N linkage is essential for biological activity, several azomethines were reported to possess remarkable antibacterial, antifungal, anticancer and diuretic activities (Supuran *et al.*, 1996). Triazoles are also important class of heterocycles because of their varied biological activities (Singh *et al.*, 2012). 1,2,3-triazoles, are five-membered, doubly unsaturated heterocycles, the ring consisting of three sequentially linked nitrogen atoms and two carbon atoms (Ajello & Cusmanos, 1940). The 1,2,3-triazole moiety has several good properties: high chemical stability (hydrolytic, oxidant, and reducing conditions), aromatic character, good hydrogen-bond-accepting ability and this moiety is relatively resistant to metabolic degradation (Holla *et al.*, 2005). Therefore, compound (I), was synthesized and its X-ray studies is reported here.

The C1–C6 and C11–C16 benzene rings of the title compound (I), (Fig. 1), make a dihedral angle of 8.9 (2)° with each other. They form dihedral angles of 52.7 (3) and 43.8 (2) ° with the N2–N4/C18/C19 propenyl ring. The C1—N1—C10—C11, C14—O1—C17—C18 and N4—C20A—C21A—C22A torsion angles are 179.7 (4), -174.2 (3) and 151.9 (14)°, respectively. The values of the bond lengths and bond angles in (I) are normal (Allen *et al.*, 1987).

In the crystal, the molecular packing of (I) is stabilized by C—H···π interactions (Table 1).

### Experimental

Reaction of 4-((1-allyl-1*H*-1,2,3-triazol-4-yl)methoxy)benzaldehyde (1.00 mmol) with 4-isopropylbenzenamine (1.00 mmol) in refluxing ethanol gave the title compound. Recrystallization from ethanol gave colourless crystals in 70% yield. Mp: 119–121 0 C. IR (KBr, cm-1):1620 (C=N). 1*H*-NMR(250 MHz, CDCl3) δ (p.p.m.): 1.11 (2CH3, d, 6H, J=7.5), 2.75 (CH, m, 1H), 4.87 (d, 2H, J=5), 5.05 (s, 2H), 5.25 (d, 2H, J=10), 5.92 (m, 1H), 6.71–7.07 (aromatic protons, m, 8H), 7.50 (H triazole, s, 1H), 8.13 (HC═N, s, 1H). 13CNMR δ (p.p.m): 23.9 (2CH3), 33.6 (CH), 52.8 (CH2—N), 61.6 (CH2—O), 114.6–145.3 (aromatic carbons and C=C triazole), 158.5 (C=N).

### Refinement

H atoms were placed in calculated positions with C—H = 0.93 - 0.98 Å and refined by using a riding model, with *U*_iso_(H) = 1.2Ueq(C) or 1.5Ueq(methyl C). The atoms of the terminal propenyl group, C20, C21 and C22, are disordered over two sites with refined occupancies of 0.714 (14) and 0.286 (14).

### Crystal data

**Table:**

C_22_H_24_N_4_O	*F*(000) = 768
*M**_r_* = 360.45	*D*_x_ = 1.214 Mg m^−^^3^
Monoclinic, *P*2_1_/*c*	Mo *K*α radiation, λ = 0.71073 Å
Hall symbol: -P 2ybc	Cell parameters from 219 reflections
*a* = 5.5885 (11) Å	θ = 3.5–21.5°
*b* = 8.3929 (18) Å	µ = 0.08 mm^−^^1^
*c* = 42.069 (9) Å	*T* = 296 K
β = 92.149 (10)°	Prism, colorless
*V* = 1971.8 (7) Å^3^	0.30 × 0.20 × 0.18 mm
*Z* = 4	

### Data collection

**Table:**

Bruker Kappa APEXII CCD diffractometer	3451 independent reflections
Radiation source: fine-focus sealed tube	1259 reflections with *I* > 2σ(*I*)
Graphite monochromator	*R*_int_ = 0.082
ω scans	θ_max_ = 25.0°, θ_min_ = 1.9°
Absorption correction: multi-scan (*SADABS*; Bruker, 2005)	*h* = −6→4
*T*_min_ = 0.982, *T*_max_ = 0.986	*k* = −9→9
13866 measured reflections	*l* = −49→49

### Refinement

**Table:**

Refinement on *F*^2^	Primary atom site location: structure-invariant direct methods
Least-squares matrix: full	Secondary atom site location: difference Fourier map
*R*[*F*^2^ > 2σ(*F*^2^)] = 0.068	Hydrogen site location: inferred from neighbouring sites
*wR*(*F*^2^) = 0.217	H-atom parameters constrained
*S* = 0.96	*w* = 1/[σ^2^(*F*_o_^2^) + (0.0893*P*)^2^] where *P* = (*F*_o_^2^ + 2*F*_c_^2^)/3
3451 reflections	(Δ/σ)_max_ < 0.001
256 parameters	Δρ_max_ = 0.20 e Å^−^^3^
7 restraints	Δρ_min_ = −0.20 e Å^−^^3^

### Special details

**Table:**

Geometry. Bond distances, angles *etc*. have been calculated using the rounded fractional coordinates. All su's are estimated from the variances of the (full) variance-covariance matrix. The cell e.s.d.'s are taken into account in the estimation of distances, angles and torsion angles
Refinement. Refinement on *F*^2^ for ALL reflections except those flagged by the user for potential systematic errors. Weighted *R*-factors *wR* and all goodnesses of fit *S* are based on *F*^2^, conventional *R*-factors *R* are based on *F*, with *F* set to zero for negative *F*^2^. The observed criterion of *F*^2^ > σ(*F*^2^) is used only for calculating -*R*-factor-obs *etc*. and is not relevant to the choice of reflections for refinement. *R*-factors based on *F*^2^ are statistically about twice as large as those based on *F*, and *R*-factors based on ALL data will be even larger.

### Fractional atomic coordinates and isotropic or equivalent isotropic displacement parameters (Å^2^)

**Table:**

	*x*	*y*	*z*	*U*_iso_*/*U*_eq_	Occ. (<1)
O1	0.6921 (5)	0.1556 (4)	0.35472 (7)	0.0741 (14)	
N1	0.0796 (6)	0.1766 (4)	0.22415 (8)	0.0634 (14)	
N2	0.7693 (6)	0.0586 (5)	0.41985 (9)	0.0879 (18)	
N3	0.8731 (6)	0.0688 (6)	0.44832 (9)	0.0939 (18)	
N4	1.1055 (6)	0.0885 (5)	0.44413 (9)	0.0762 (16)	
C1	−0.0186 (7)	0.1560 (5)	0.19307 (10)	0.0581 (17)	
C2	−0.2092 (7)	0.2503 (6)	0.18440 (11)	0.0708 (19)	
C3	−0.3158 (8)	0.2422 (6)	0.15464 (12)	0.084 (2)	
C4	−0.2360 (8)	0.1385 (6)	0.13217 (11)	0.0717 (19)	
C5	−0.0502 (9)	0.0411 (6)	0.14117 (11)	0.0805 (19)	
C6	0.0617 (7)	0.0478 (6)	0.17103 (11)	0.0763 (19)	
C7	−0.3528 (10)	0.1319 (8)	0.09917 (12)	0.106 (3)	
C8	−0.1850 (11)	0.1621 (7)	0.07339 (12)	0.137 (3)	
C9	−0.4985 (10)	−0.0154 (8)	0.09435 (12)	0.132 (3)	
C10	0.2749 (8)	0.1146 (5)	0.23238 (10)	0.0681 (19)	
C11	0.3876 (7)	0.1292 (5)	0.26388 (10)	0.0577 (17)	
C12	0.2894 (7)	0.2200 (5)	0.28752 (10)	0.0633 (17)	
C13	0.3977 (7)	0.2279 (5)	0.31714 (10)	0.0653 (19)	
C14	0.6026 (7)	0.1427 (5)	0.32409 (10)	0.0578 (17)	
C15	0.7072 (7)	0.0562 (5)	0.30096 (10)	0.0638 (17)	
C16	0.5967 (8)	0.0500 (5)	0.27125 (10)	0.0683 (19)	
C17	0.8850 (7)	0.0529 (6)	0.36388 (9)	0.0683 (19)	
C18	0.9390 (7)	0.0723 (5)	0.39809 (10)	0.0618 (18)	
C19	1.1516 (7)	0.0906 (5)	0.41344 (10)	0.0684 (19)	
C20A	1.2557 (18)	0.0919 (15)	0.4733 (2)	0.083 (3)	0.714 (14)
C21A	1.251 (2)	0.254 (2)	0.4850 (4)	0.138 (7)	0.714 (14)
C22A	1.272 (2)	0.3163 (18)	0.5105 (3)	0.203 (8)	0.714 (14)
C21B	1.341 (6)	0.263 (6)	0.4899 (12)	0.138 (7)	0.286 (14)
C22B	1.120 (5)	0.342 (4)	0.4916 (8)	0.203 (8)	0.286 (14)
C20B	1.318 (4)	0.118 (5)	0.4674 (6)	0.083 (3)	0.286 (14)
H2	−0.26800	0.32180	0.19910	0.0850*	
H3	−0.44500	0.30850	0.14950	0.1010*	
H8A	−0.08690	0.25270	0.07880	0.2060*	
H8B	−0.27430	0.18290	0.05390	0.2060*	
H8C	−0.08510	0.07040	0.07080	0.2060*	
H9A	−0.39530	−0.10690	0.09540	0.1980*	
H5	0.00360	−0.03290	0.12660	0.0970*	
H6	0.18930	−0.01960	0.17620	0.0910*	
H7	−0.46740	0.22030	0.09820	0.1270*	
H12	0.14840	0.27620	0.28320	0.0760*	
H13	0.33210	0.29140	0.33270	0.0790*	
H15	0.85030	0.00240	0.30520	0.0760*	
H16	0.66660	−0.01020	0.25550	0.0820*	
H17A	1.02500	0.07910	0.35200	0.0820*	
H17B	0.84150	−0.05690	0.35930	0.0820*	
H19	1.30020	0.10220	0.40450	0.0820*	
H20A	1.19370	0.01930	0.48890	0.1000*	0.714 (14)
H20B	1.41820	0.06050	0.46890	0.1000*	0.714 (14)
H21A	1.22690	0.32730	0.46860	0.1660*	0.714 (14)
H22A	1.29690	0.25460	0.52870	0.2430*	0.714 (14)
H22B	1.26220	0.42660	0.51220	0.2430*	0.714 (14)
H9B	−0.58050	−0.01150	0.07390	0.1980*	
H9C	−0.61350	−0.02290	0.11070	0.1980*	
H10	0.35350	0.05510	0.21730	0.0820*	
H20C	1.45990	0.11910	0.45480	0.1000*	0.286 (14)
H20D	1.33110	0.02440	0.48090	0.1000*	0.286 (14)
H21B	1.48080	0.29430	0.50080	0.1660*	0.286 (14)
H22C	0.98690	0.30480	0.48000	0.2430*	0.286 (14)
H22D	1.10720	0.43250	0.50430	0.2430*	0.286 (14)

### Atomic displacement parameters (Å^2^)

**Table:**

	*U*^11^	*U*^22^	*U*^33^	*U*^12^	*U*^13^	*U*^23^
O1	0.077 (2)	0.081 (3)	0.063 (2)	0.0161 (18)	−0.0145 (16)	−0.0069 (16)
N1	0.057 (2)	0.072 (3)	0.061 (2)	0.001 (2)	−0.0013 (19)	0.0060 (19)
N2	0.047 (2)	0.146 (4)	0.070 (3)	0.000 (2)	−0.005 (2)	0.016 (3)
N3	0.052 (2)	0.159 (4)	0.071 (3)	0.003 (3)	0.005 (2)	0.020 (3)
N4	0.049 (2)	0.111 (3)	0.068 (3)	0.002 (2)	−0.007 (2)	0.001 (2)
C1	0.054 (3)	0.066 (3)	0.054 (3)	−0.010 (3)	−0.001 (2)	0.004 (2)
C2	0.056 (3)	0.081 (4)	0.075 (3)	0.009 (3)	−0.004 (2)	−0.013 (3)
C3	0.071 (3)	0.096 (4)	0.084 (4)	0.023 (3)	−0.014 (3)	−0.009 (3)
C4	0.068 (3)	0.080 (4)	0.066 (3)	−0.004 (3)	−0.011 (3)	0.008 (3)
C5	0.078 (3)	0.092 (4)	0.071 (3)	0.008 (3)	−0.002 (3)	−0.014 (3)
C6	0.073 (3)	0.085 (4)	0.070 (3)	0.015 (3)	−0.009 (3)	−0.006 (3)
C7	0.112 (4)	0.122 (6)	0.082 (4)	−0.003 (4)	−0.022 (4)	−0.004 (3)
C8	0.184 (6)	0.154 (6)	0.073 (4)	−0.071 (5)	−0.010 (4)	0.010 (4)
C9	0.112 (4)	0.187 (7)	0.096 (4)	−0.044 (5)	−0.020 (3)	−0.008 (4)
C10	0.069 (3)	0.076 (4)	0.059 (3)	0.001 (3)	0.000 (2)	−0.003 (2)
C11	0.052 (3)	0.067 (3)	0.054 (3)	−0.002 (2)	−0.001 (2)	0.006 (2)
C12	0.059 (3)	0.072 (3)	0.059 (3)	0.010 (2)	0.002 (2)	0.008 (3)
C13	0.066 (3)	0.070 (4)	0.060 (3)	0.008 (3)	0.002 (2)	0.002 (2)
C14	0.056 (3)	0.061 (3)	0.056 (3)	−0.003 (2)	−0.004 (2)	0.005 (2)
C15	0.054 (3)	0.069 (3)	0.068 (3)	0.009 (2)	−0.002 (2)	−0.005 (3)
C16	0.068 (3)	0.075 (4)	0.062 (3)	0.004 (3)	0.003 (2)	−0.011 (3)
C17	0.053 (3)	0.077 (4)	0.074 (3)	0.001 (3)	−0.011 (2)	−0.002 (3)
C18	0.045 (2)	0.080 (4)	0.060 (3)	0.001 (2)	−0.005 (2)	0.005 (3)
C19	0.051 (3)	0.091 (4)	0.063 (3)	−0.009 (3)	−0.001 (2)	0.000 (3)
C20A	0.044 (5)	0.140 (7)	0.065 (5)	−0.006 (5)	0.002 (4)	0.014 (5)
C21A	0.142 (16)	0.172 (9)	0.094 (10)	0.010 (11)	−0.082 (8)	−0.019 (7)
C22A	0.227 (14)	0.216 (13)	0.158 (13)	0.046 (10)	−0.088 (10)	−0.053 (10)
C21B	0.142 (16)	0.172 (9)	0.094 (10)	0.010 (11)	−0.082 (8)	−0.019 (7)
C22B	0.227 (14)	0.216 (13)	0.158 (13)	0.046 (10)	−0.088 (10)	−0.053 (10)
C20B	0.044 (5)	0.140 (7)	0.065 (5)	−0.006 (5)	0.002 (4)	0.014 (5)

### Geometric parameters (Å, º)

**Table:**

O1—C14	1.369 (5)	C21B—C22B	1.41 (5)
O1—C17	1.422 (5)	C2—H2	0.9300
N1—C1	1.410 (5)	C3—H3	0.9300
N1—C10	1.246 (6)	C5—H5	0.9300
N2—N3	1.314 (5)	C6—H6	0.9300
N2—C18	1.348 (5)	C7—H7	0.9800
N3—N4	1.328 (5)	C8—H8A	0.9600
N4—C19	1.326 (6)	C8—H8B	0.9600
N4—C20A	1.461 (10)	C8—H8C	0.9600
N4—C20B	1.53 (2)	C9—H9A	0.9600
C1—C2	1.366 (6)	C9—H9B	0.9600
C1—C6	1.385 (6)	C9—H9C	0.9600
C2—C3	1.368 (7)	C10—H10	0.9300
C3—C4	1.372 (7)	C12—H12	0.9300
C4—C5	1.364 (7)	C13—H13	0.9300
C4—C7	1.513 (7)	C15—H15	0.9300
C5—C6	1.383 (7)	C16—H16	0.9300
C7—C8	1.482 (8)	C17—H17A	0.9700
C7—C9	1.490 (9)	C17—H17B	0.9700
C10—C11	1.451 (6)	C19—H19	0.9300
C11—C12	1.383 (6)	C20A—H20A	0.9700
C11—C16	1.370 (6)	C20A—H20B	0.9700
C12—C13	1.366 (6)	C20B—H20C	0.9700
C13—C14	1.372 (6)	C20B—H20D	0.9700
C14—C15	1.363 (6)	C21A—H21A	0.9300
C15—C16	1.374 (6)	C21B—H21B	0.9300
C17—C18	1.468 (6)	C22A—H22A	0.9300
C18—C19	1.340 (6)	C22A—H22B	0.9300
C20A—C21A	1.45 (2)	C22B—H22C	0.9300
C20B—C21B	1.54 (6)	C22B—H22D	0.9300
C21A—C22A	1.20 (2)		
			
C14—O1—C17	117.0 (3)	C8—C7—H7	106.00
C1—N1—C10	120.9 (4)	C9—C7—H7	106.00
N3—N2—C18	108.4 (3)	C7—C8—H8A	109.00
N2—N3—N4	106.7 (3)	C7—C8—H8B	109.00
N3—N4—C19	111.0 (3)	C7—C8—H8C	109.00
N3—N4—C20A	115.2 (5)	H8A—C8—H8B	109.00
N3—N4—C20B	132.4 (9)	H8A—C8—H8C	109.00
C19—N4—C20A	133.7 (5)	H8B—C8—H8C	110.00
C19—N4—C20B	116.5 (9)	C7—C9—H9A	109.00
N1—C1—C2	116.7 (4)	C7—C9—H9B	109.00
N1—C1—C6	125.2 (4)	C7—C9—H9C	109.00
C2—C1—C6	118.1 (4)	H9A—C9—H9B	110.00
C1—C2—C3	121.6 (4)	H9A—C9—H9C	109.00
C2—C3—C4	121.4 (4)	H9B—C9—H9C	110.00
C3—C4—C5	116.9 (4)	N1—C10—H10	118.00
C3—C4—C7	121.0 (4)	C11—C10—H10	118.00
C5—C4—C7	122.1 (5)	C11—C12—H12	120.00
C4—C5—C6	122.8 (4)	C13—C12—H12	120.00
C1—C6—C5	119.2 (4)	C12—C13—H13	120.00
C4—C7—C8	113.7 (5)	C14—C13—H13	120.00
C4—C7—C9	111.7 (5)	C14—C15—H15	121.00
C8—C7—C9	113.6 (5)	C16—C15—H15	121.00
N1—C10—C11	124.3 (4)	C11—C16—H16	119.00
C10—C11—C12	122.2 (4)	C15—C16—H16	119.00
C10—C11—C16	120.2 (4)	O1—C17—H17A	110.00
C12—C11—C16	117.6 (4)	O1—C17—H17B	110.00
C11—C12—C13	120.6 (4)	C18—C17—H17A	110.00
C12—C13—C14	120.4 (4)	C18—C17—H17B	110.00
O1—C14—C13	115.6 (4)	H17A—C17—H17B	108.00
O1—C14—C15	124.2 (4)	N4—C19—H19	127.00
C13—C14—C15	120.2 (4)	C18—C19—H19	127.00
C14—C15—C16	118.6 (4)	N4—C20A—H20A	110.00
C11—C16—C15	122.5 (4)	N4—C20A—H20B	110.00
O1—C17—C18	108.9 (3)	C21A—C20A—H20A	110.00
N2—C18—C17	122.2 (4)	C21A—C20A—H20B	110.00
N2—C18—C19	108.4 (4)	H20A—C20A—H20B	109.00
C17—C18—C19	129.1 (4)	C21B—C20B—H20C	106.00
N4—C19—C18	105.5 (4)	C21B—C20B—H20D	106.00
N4—C20A—C21A	106.7 (10)	H20C—C20B—H20D	106.00
N4—C20B—C21B	124 (3)	N4—C20B—H20D	106.00
C20A—C21A—C22A	135.6 (16)	N4—C20B—H20C	106.00
C20B—C21B—C22B	111 (3)	C20A—C21A—H21A	112.00
C1—C2—H2	119.00	C22A—C21A—H21A	113.00
C3—C2—H2	119.00	C20B—C21B—H21B	125.00
C2—C3—H3	119.00	C22B—C21B—H21B	124.00
C4—C3—H3	119.00	C21A—C22A—H22A	120.00
C4—C5—H5	119.00	C21A—C22A—H22B	120.00
C6—C5—H5	119.00	H22A—C22A—H22B	120.00
C1—C6—H6	120.00	C21B—C22B—H22C	120.00
C5—C6—H6	120.00	C21B—C22B—H22D	120.00
C4—C7—H7	106.00	H22C—C22B—H22D	120.00
			
C17—O1—C14—C15	−10.0 (6)	C5—C4—C7—C9	71.5 (6)
C17—O1—C14—C13	171.9 (4)	C3—C4—C7—C9	−107.5 (6)
C14—O1—C17—C18	−174.2 (3)	C7—C4—C5—C6	178.7 (5)
C1—N1—C10—C11	−179.7 (4)	C5—C4—C7—C8	−58.6 (7)
C10—N1—C1—C6	11.9 (6)	C3—C4—C7—C8	122.3 (6)
C10—N1—C1—C2	−168.6 (4)	C4—C5—C6—C1	0.9 (7)
N3—N2—C18—C19	0.3 (5)	N1—C10—C11—C16	176.6 (4)
C18—N2—N3—N4	−0.1 (5)	N1—C10—C11—C12	−2.5 (7)
N3—N2—C18—C17	174.9 (4)	C10—C11—C16—C15	−178.2 (4)
N2—N3—N4—C20A	−176.1 (6)	C10—C11—C12—C13	178.4 (4)
N2—N3—N4—C19	−0.2 (6)	C12—C11—C16—C15	1.0 (6)
N3—N4—C20A—C21A	−84.7 (9)	C16—C11—C12—C13	−0.7 (6)
N3—N4—C19—C18	0.3 (5)	C11—C12—C13—C14	−1.6 (6)
C20A—N4—C19—C18	175.2 (8)	C12—C13—C14—C15	3.6 (6)
C19—N4—C20A—C21A	100.6 (9)	C12—C13—C14—O1	−178.1 (4)
N1—C1—C6—C5	−179.4 (4)	C13—C14—C15—C16	−3.3 (6)
C2—C1—C6—C5	1.1 (7)	O1—C14—C15—C16	178.6 (4)
N1—C1—C2—C3	178.9 (4)	C14—C15—C16—C11	1.0 (6)
C6—C1—C2—C3	−1.6 (7)	O1—C17—C18—N2	53.0 (6)
C1—C2—C3—C4	0.1 (7)	O1—C17—C18—C19	−133.6 (5)
C2—C3—C4—C7	−179.2 (5)	N2—C18—C19—N4	−0.4 (5)
C2—C3—C4—C5	1.8 (7)	C17—C18—C19—N4	−174.5 (4)
C3—C4—C5—C6	−2.3 (7)	N4—C20A—C21A—C22A	151.9 (14)

### Hydrogen-bond geometry (Å, º)

Cg1 and Cg3 are the centroids of the N2–N4/C18/C19 1H-1,2,3-triazole and C11–C16 benzene rings, respectively.

**Table:**

*D*—H···*A*	*D*—H	H···*A*	*D*···*A*	*D*—H···*A*
C2—H2···*Cg*3^i^	0.93	2.96	3.752 (5)	144
C8—H8*A*···*Cg*1^ii^	0.96	2.80	3.678 (6)	153

Symmetry codes: (i) −*x*, *y*+1/2, −*z*+1/2; (ii) −*x*+1, *y*+1/2, −*z*+1/2.
